# Principles of Judo Training as an Organised Form of Physical Activity for Children

**DOI:** 10.3390/ijerph19041929

**Published:** 2022-02-09

**Authors:** Monika Kowalczyk, Małgorzata Zgorzalewicz-Stachowiak, Wiesław Błach, Maciej Kostrzewa

**Affiliations:** 1 Department of Health Prophylaxis, University of Medical Sciences, 61-701 Poznan, Poland; neuro@ump.edu.pl; 2 Faculty of Physical Education & Sport, University School of Physical Education, 51-612 Wroclaw, Poland; wieslaw.judo@wp.pl; 3 Institute of Sport Science, The Jerzy Kukuczka Academy of Physical Education in Katowice, 40-065 Katowice, Poland

**Keywords:** judo, physical education and training, sports injuries, child

## Abstract

When organising judo training for children, it is essential to ensure maximum safety, and use an appropriate training methodology adapted to the age of the youngest judo athletes. This paper aims to review the current literature containing judo training principles and safety-related considerations for preschool (4–6 years) and school-age (7–12 years) children as an organised physical activity. Data were collected until October 2021 from eight international scientific databases (PubMed, Scopus, UpToDate, Web of Science, Cochrane, EBSCOhost, ScienceDirect, Google Scholar). In the overviews, we found various times and frequencies of judo training for children. In preschool, the training time was 30–60 min with a frequency of 2–3 times per week, whereas in the school-age group, the training time was extended from 45 to 90 min 3–4 times per week. The most common injuries included upper arm injuries, followed by those of the lower limbs. In the future, it would be an advantage to systematise the methodology of judo training as an organised form of physical activity that can complement the daily dose of exercises recommended by the World Health Organization for maintaining children’s general health.

## 1. Introduction

The sedentary lifestyle of children is a serious problem in the 21st century. Fewer and fewer children participate in spontaneous outdoor games, and children are spending more time in front of multimedia screens. To maintain the psychophysical health of children aged 5–17 years, the World Health Organization (WHO) recommends physical activity (PA) for an average of 60 min per day at moderate to intense levels [1]. In the Polish curriculum, the weekly time allocated to physical education in the early school period (ages 7–10) is three classes per week for 45 min; in the school period (ages ten and above), it is four times per week for 45 min [2]. As a result, the PA time in school settings is insufficient to meet children’s movement needs. Therefore, in addition to educational establishments, it is mainly the parents who should take care of their children’s physical development.

Martial arts are now becoming increasingly popular around the world. These include judo, which is currently practised by millions of people. From the point of view of parents who enrol their children in training, judo is a PA that affects all aspects (physical, psychological and social) of practitioners’ health [3]. Judo training can occur as an additional organised form of PA outside school, sports clubs, or physical education lessons. The International Judo Federation (IJF), taking into account the health and educational advantages of judo, created the “Judo in Schools” programme, which is available worldwide [4]. Its main goal is to present this martial art to as many children as possible, especially those aged 4–12, and to encourage them to train regularly. In addition, the programme’s creators would like judo to become part of the core curriculum of physical education in schools globally.

### History of Judo

Judo is a martial art created in Japan by Professor Jigoro Kano in the late 19th century. The word judo in Japanese translates as “the gentle way”. Kano developed his techniques from various Japanese jujitsu schools [5]. Moreover, he described judo’s educational and upbringing values based on the fundamental principles of Seiryoku-Zenyo and Jita-Kyoei [6,7,8,9]. According to these principles, the purpose of practising this martial art is to learn how to attack and defend with effective use of the body and mind energy (Seiryoku-Zenyo). Kano described it as the art of executing an effective technique during a fight appropriate to the action, where fighting is a learning process rather than the end in itself.

Furthermore, he emphasised the importance of self-improvement during training, resulting in mutual social benefits for all fellow practitioners on and off the mat (Jita-Kyoei). According to judo’s philosophy, doing good, respect, harmony, discipline, and cooperation are essential in the training room and everyday life [10]. As a teenager, Jigoro Kano himself experienced violence from older classmates at jujitsu school [7]. To eliminate aggression and the use of force towards the weaker, he introduced white judo suits (judogi, equal for all), a division into belts (from white as a student’s kyu to black as a master’s dan). Kano also pointed out that fellow practitioners should be of similar weight and age to provide some protection for novices. In judo’s etiquette, based on Japan’s cultural traditions, he also included mutual bows to reflect respect for the other people training on the mat.

Following Jigoro Kano’s philosophy “to the general good”, judo training connects educational, social and health benefits [11]. De Crée [12] suggests that in training children, the initial intention of the founder of judo was to foster a progressive and complete form of physical and mental education. Kano’s assumption through judo was the possibility of using the power of mind and body most effectively. Its training cultivates the body and spirit through the practice of attack and defence, and the essence of this principled moral code is learning through self-awareness. Judo has now evolved into a sport that focuses more on competition and winning. Therefore, it is essential to emphasise the fundamental principles of judo (Seiryoku-Zenyo and Jita-Kyoei), aiming at perfecting oneself and benefiting from life [13]. The educational benefit of judo is a value that should be cultivated among judo practitioners, especially the youngest.

Judo competitions are divided into age and weight categories [14]. In seniors (over the age of 20), there are seven weight categories for men and women. In the younger age categories, due to the biological development of children and adolescence, there are more designated weight categories according to the sports regulations of a given country, based on IJF rules. In Poland, children from 11 years of age (calendar age) can compete in regional competitions [14,15]. Children from the age of 14 (calendar year) can participate in national competitions. They must have a licence from the Polish Judo Association and up-to-date medical examinations.

Practitioners’ clothing consists of judogi—trousers, jacket and belt. During official sports competitions, one competitor currently wears a white judogi and their opponent wears a blue one. The differently coloured judogi make it easier for the judges to distinguish and score the contestants during a fight.

This study aimed to review the current literature on the practical organisation and conduct of judo training for healthy children of preschool and school-age. The review analysed the purpose, forms and methods of training for children. The authors also took note of safety aspects and the risk of injury during such classes.

## 2. Materials and Methods

Until October 2021, publications were collected from eight international scientific databases (PubMed, Scopus, UpToDate, Web of Science, Cochrane, EBSCOhost, ScienceDirect, Google Scholar). Papers in English or Polish, dating from the 1st of January 2009 onwards, were considered. During the search of the relevant literature, “judo” in combination with the following terms: martial arts, children, young, youth, girls, boys, training, techniques, injuries, physical activity, was used. The analysis involved publications that presented forms and methods of judo-specific training for healthy preschool children (4–6 years old) and school-age children (7–12 years old) and the risk of injury. The references sections of these studies were also screened. There was no information found about injuries in judo in the 4–12 years old age group. Therefore, the available reports about injuries in children and adolescents up to 18 years of age were taken into account. Original research and reviews related to the presented topic were qualified.

## 3. Results

### 3.1. When Can Children Start Training Judo?

From the available literature, it can be seen that children start training from preschool age. Based on the available publications, it can be concluded that the youngest participants of judo lessons in Poland are between four and six years old [3,16,17]. Similarly, in her work, Demiral [18] proposes a primary judo curriculum for children aged 4–6. In the publication by Garcia et al. [19], describing a training programme of judo techniques for children, the authors indicate that of all children starting judo, 73% are aged 4–8, the majority of whom began training at the age of five or six (17.7% and 22.9%, respectively).

### 3.2. What Is the Recommended Duration and Frequency of Training?

Sterkowicz-Przybycień et al. [3] suggest that the duration of judo lessons for practitioners aged 4–6 should be 30–35 min twice per week. In studies by Walaszek et al. [16,17], children aged 5–6 exercised for 35–45 min 2–3 times per week. Neofit [20] describes recommendations based on feedback and coaching experience and suggests that judo training for a group of 4–6-year-olds should take place 2–3 times per week for 45 min. In Krustolovic et al.’s [21] study, children of an average age of six practised judo three times per week for 45 min, and in Djordjevic et al.’s [22] research, boys of an average age of six practised judo three times per week for 60 min. School practitioners in the study by Triki et al. [23], at the age of ten, trained in judo four times per week for 90 min. In Ludyga et al. [24], judokas with an average age of ten practised twice per week for 60 min. The judokas in Jankowicz-Szymanska et al.’s [25] and Maślinski et al.’s [26] studies were aged 11–12 and received judo lessons three times per week for 90 min. In the recent review of studies on the effectiveness of judo exercise in children, Gutierrez-Garcia et al. [10] point to different class methodologies for children aged 5–12. Their class duration was 35–90 min, and the frequency was between two and three times per week.

Throughout the judo training methodology, it is emphasised that activities should be adapted to children’s abilities and psychomotor needs, and relate to their age. The Canadian Long Term Athlete Development (LTAD) judo training programme is based on children’s so-called motor development windows [27]. The programme is designed to select athletes for practicing judo competitively and to achieve results at major sporting events. The first window of the programme covers the development of speed capabilities and occurs at 6–8 years for girls and 7–9 years for boys. The next window focuses on agility skills occurring in girls aged 8–11 and boys 9–12. For judoka under 11 years, it is recommended that the duration of training is 60–90 min 2–3 times per week; for those under the age of 13, it is suggested that training takes place 3–4 times per week for 60–90 min.

### 3.3. Purpose of the Training

In the early years, training should focus primarily on developing general physical fitness (coordination, speed, agility, efficiency and balance), learning how to safely break falls, and basic judo techniques [18,19,28,29].

The teaching of judo elements begins with learning how to fall (Ukemi), techniques on the ground (Ne-waza), through to learning grappling techniques (Katame-waza) and those when standing (Tachi-waza). The grappling group includes bracing, joint locking and choking techniques. Bracing techniques are taught first, and for safety reasons, joint locking and choking techniques are only allowed in sports competitions much later (in Poland from the age of 14 calendar years). Next, throwing techniques (Nage-waza) in the standing position are introduced (Tachi-waza). When practitioners have reached a sufficient level of fitness and skills, combat (Randori) elements are performed.

Moreover, training should not only focus on the development of physical attributes. Jigoro Kano, the founder of the first Kodokan School, also emphasised the formation of positive character traits such as discipline, responsibility and the ability to control emotions through training. According to the principles of Seiryoku-Zenyo and Jita-Kyoei, through judo, one should develop respect and modesty towards fellow practitioners, teachers, and also towards general social contacts [7,8].

### 3.4. Forms of Training

We found no standardised description in the literature of the forms of judo training in children as an organised form of activity. It would be beneficial if it involved a long-term evaluation of its effectiveness and influence on health. Currently, teachers choose these forms mainly based on their own knowledge and experience, adjusting the programme to the age and abilities of the persons exercising. Van Kooten [27] points out that in teaching judo children under 12 coaches should avoid “specialisation in judo, negative competitive experiences, and comparison with other children, while all activities should take the form of games”.

Sterkowicz-Przybycień et al. [3], describing the curriculum for 4–6-year-olds, indicate that the main aim is to develop the movement skills through gymnastic and general development exercises. Moreover, the youngest children, when starting their training, learn proper judo bows, natural and defensive postures (Shisei), movement on the mat and, above all, falling on the mat (Ukemi). In the following stages, they learn basic holds (Hon-kesa-gatame, Yoko-shiho-gatame and Kami-shiho-gatame), body rotations (Tai-sabaki) and basic throws (O-goshi and O-soto-gari).

In their paper, Krustolovic et al. [21] present a judo programme for children with an average age of seven. In their nine-month programme, each 45-min workout is divided into three stages: warm-up (14–19 min), main part (25 min) and cool-down (5–8 min). The warm-up is based on general development, gymnastics and stretching exercises. In the main part, for 15 min, the following judo elements are practised: postures (Shisei), grasping the judogi (Kumi-kata), moving on the mat (Shintai), falls (Ukemi), throws (Nage-waza) and techniques on the ground (Ne-waza), and fighting (Randori). The next ten minutes are devoted to selecting games connected with learning judo techniques. The end of the session is a phase of calming down, relaxation and stretching.

A similar training scheme for children of an average age of ten for 45 to 60 min is presented by Missawi et al. [30]. The warm-up lasts 15–20 min with low-intensity games and stretching exercises, followed by the central part of the training session lasting 20–25 min. At this stage of training, pads (Ukemi), throws and combinations of these techniques (Uchi-komi, Renraku-waza) are perfected, followed by training fights (five minutes). The last 5–10 min are devoted to active regeneration. Ludyga et al. [24] also present a programme for school children beginning judo at an average age of ten. Each judo session consists of playful physical fitness exercises and judo techniques, including falling techniques and Randori (fighting).

Garcia et al. [19] propose a judo-teaching programme for children based on a survey conducted among 911 judo teachers from 19 countries. The trainers’ responses were based on their knowledge and experience. The authors’ most important point is adapting technical elements to the children’s age and developmental abilities. The implementation of particular judo techniques should be based on two criteria: the safety of the fellow practitioner (Uke) and the ease of executing the throw. The teacher should observe if the person performing the throw (Tori) controls their and the Uke’s movements while falling. The authors suggest that the examination for the first white belt at the age of about six should include the ability to perform pads and move on the mat. Then, at about seven, trainers can start teaching children for their yellow belt using the most comfortable throws such as O-soto-gari, O-goshi, De-Ashi-barai, and Ko-soto-gari. The colours of the kyu training belts in Europe range from white through yellow, orange, green, blue and brown. After mastering the most difficult brown belt techniques, the student can take the master dan black belt exam. The authors emphasise the obtaining of a black belt and adequate physical fitness and technical skills. There are also minimum age criteria to maintain the child’s safety. In Poland and Spain, the minimum age is 16 years old.

Games implemented in play are considered an exciting way to teach judo to children. This free activity has certain rules, and is performed in a specific place and time. The ending of games is not determined and allows learning to be adapted to solving problem situations during the game. The game may be used to introduce judo techniques separately or judo in its entirety with the ability to fight [31]. Children very much enjoy these games as they are motivating and receive praise when they correctly perform technique. Lukanova [32] and Masenko [33] describe judo training based on games and activities. According to these authors, training based on selected games should last between 24 and 45 min in total. However, short task games based on performing a single exercise should not be longer than 30–60 s, with a maximum of 3–5 repetitions. Simultaneously, the duration of longer games with a more complex exercise structure is 3–10 min with a similar number of repetitions [32,33]. In a recent report, Pereira et al. [31] present the systematisation of games through a network of complex connections. According to the authors, the proposed game system covers spheres from A to F, consisting of various functional units. The number of connections between the spheres and units of a given game affect its complexity and difficulty level. The presented network can be used for different learning levels, from judo beginners to professional participants. The authors indicate that such a teaching system may positively affect the teaching of judo techniques and their use in combat. Appropriately selected game elements can enrich movements and develop general physical fitness, judo tactics and technique elements. They also provide an introduction to the development of sports competition skills, which can be useful at a later stage of training.

### 3.5. Children Safety during Training

Trainers and the parents of children taking part in judo training must know about the associated injury risks. In the limited literature, the incidence of injuries was only observed together in the wider group aged under 18 [34,35]. According to Pocceco et al. [34] and Demorest et al. [35], reviews concern injuries for the whole group of those aged 5–17. The available data do not show any correlation between the ages of the children training and the incidence of injuries. The authors report that injuries are often associated with incorrect throwing and falling techniques and when there is too much of a difference in the body weights of those training. Moreover, they mention that the most common injuries are contusions, fractures, and sprains. These mainly affect the shoulder, elbow and wrist in the upper limb, and the foot and ankle in the lower limb. Demorest et al. [35] suggest that the more frequent upper limb injuries in judo, compared to other martial arts such as karate and taekwondo, where kicks are mainly performed, may occur due to judo-specificity. Judogi grabbing when throwing and falling on the mat are associated with more significant work of the upper limbs when performing judo techniques. Unfortunately, there are no data on related risk factors and judo injuries in younger children aged 4–12 years. The literature does not describe any injuries for the age group discussed that correlate with the length, intensity or difficulty of the techniques performed, or with the progress of the children.

In preventing injuries, the essential principle in judo training should be to ensure the maximum safety of the practitioners. According to Pocecco et al.’s [34] review, the trainer’s qualifications, knowledge and experience in teaching this sport to children are of primary importance in injury prevention. The crucial element is to teach how to perform proper falling techniques, how to fall during throws and how to correctly execute throws. It is also essential to provide adequate, comprehensive physical development and prevent adepts from competing too early, that is, participating in sports competitions. Further, the mat intended for exercise should be of the appropriate quality and firmness to ensure proper cushioning for falls.

## 4. General Recommendations

Within this review, we found various methodologies for teaching judo to children. Some authors [3,16,19] suggest that judo classes could be started at the preschool age of between four and six years. Furthermore, analysing the available data [3,16,17,20,21] on the length and frequency of the training itself, the training time for preschool children (4–6 years) is between 30 min and up to even 60 min with a frequency of 2–3 times per week. At school age, authors [23,24,25,26] recommend extending the time from 45 to 90 min with a frequency of 3–4 times per week.

Numerous studies [5,19,21,27,28,29] conclude with similar aims when teaching judo as a way of developing physical fitness, learning judo elements and forming pro-social attitudes based on the original assumptions of the creator of judo, Jigoro Kano. However, the forms and methods of judo training are varied, depending on the teacher’s plan [19]. Sterkowicz-Przybycień et al. [3] focus on developing physical fitness and learning basic judo elements such as falls, bracing techniques and basic throws (O-goshi and O-soto-gari). By comparison, Garcia et al. [19] propose a programme in which training children up to the age of six can develop general fitness, learn break-falls and how to move on the mat. They suggest starting by learning the most straightforward throws (O-soto-gari, O-goshi, De-Ashi-barai and Ko-soto-gari) from the age of seven. The description of the forms of training for the school groups in Krustolovic et al. [21] and Missawi et al. [30], despite the similar division of classes into three parts, differ in their duration. The warm-up time as an introduction to the class varies between 14 and 20 min. The main part of the training, dedicated to learning judo and training fight elements, last 20–25 min. Relaxation, calming down and stretching are performed in the class’s last 5–10 min.

Smaruj et al. [36] and Kostrzewa et al. [37] mention strength, correct posture, effective movements and balance as critical elements in judo. Due to the activity’s complexity, judo training can also be considered as a tool for increasing the strength of postural muscles, which should be taken into account when planning training for children.

Additionally, movement games and physical activities with judo elements are recommended as an exciting form of training for children [31,32,33]. The advantage of games and activities are that they simultaneously develop physical fitness while teaching the elements of judo, encourage the solving of problem situations during a game, and provide pleasure and satisfaction.

An important aspect during training, emphasised in the literature, is to provide an adequate safety level, for which the judo teacher is responsible [34]. Their professional qualifications and experience, and the equipment in the training halls, are important. Although, worldwide, children are more willingly practice, the risk of injuries should be taken into account when starting training. According to the reviews by Pocceco et al. [34] and Demorest et al. [35], the most common injuries are contusions, fractures or sprains of the upper limb, but these occur less often in the lower limb. In preventing injuries, it is essential to conduct regular medical check-ups that confirm no contraindications for practising judo and allow children to compete in sport according to the sports medicine regulations in force in a given country.

## 5. Conclusions

The advantage of judo is that it is a sport for participants of all ages and the cost of preparing a practice hall is low, with only mats being essential. However, although judo has existed for over 100 years, there are insufficient training guidelines for the youngest children, combined with assessments of its effectiveness and health benefits. The available publications show some methodological variation in this respect. Choosing a teaching programme is up to the teachers, based on their education, preparation and experience of working with children. It would be advantageous to create a relevant literature database of judo methodology for children with long-term observations of its effectiveness and influence on general health, which could be used primarily by young and less experienced teachers. In Poland, coaching can be provided by people who have adequate knowledge of judo, a minimum qualification of 1st Kyu (brown belt), have completed an appropriate training course or graduated in this field, and have registered with the Polish Judo Association and obtained a licence [15]. These people usually have a rich past as competitive athletes but are just beginning to gather coaching experience, generally by training the youngest children. It would be beneficial to systematise the forms and methods of judo training teachers. Additionally, as a supplement to the existing knowledge, an exciting research direction would also be assessing injuries in children aged 4–12 years, where activities are more recreational and there is less pressure on achieving sports results.

Judo, as a structured PA with the appropriate frequency, intensity and duration of exercise for preschool and school-age children, can supplement the WHO’s recommendation for the amount of daily exercise to be undertaken. This is important for combating the sedentary lifestyles of this age group and the related rising tide of obesity.
